# A Closed Posterior Ankle Dislocation Without Overt Associated Fracture as a Result of a Trampoline Park Injury

**DOI:** 10.1016/j.acepjo.2025.100149

**Published:** 2025-04-24

**Authors:** Kahra Nix, Caroline Gosser, John-Matthew Ang, Daniel Perling, Melissa Platt

**Affiliations:** 1 Department of Emergency Medicine, University of Louisville, Louisville, Kentucky, USA; 2 University of Louisville Medical School, Louisville, Kentucky, USA

## 1 Patient Presentation

**Figure 1:**
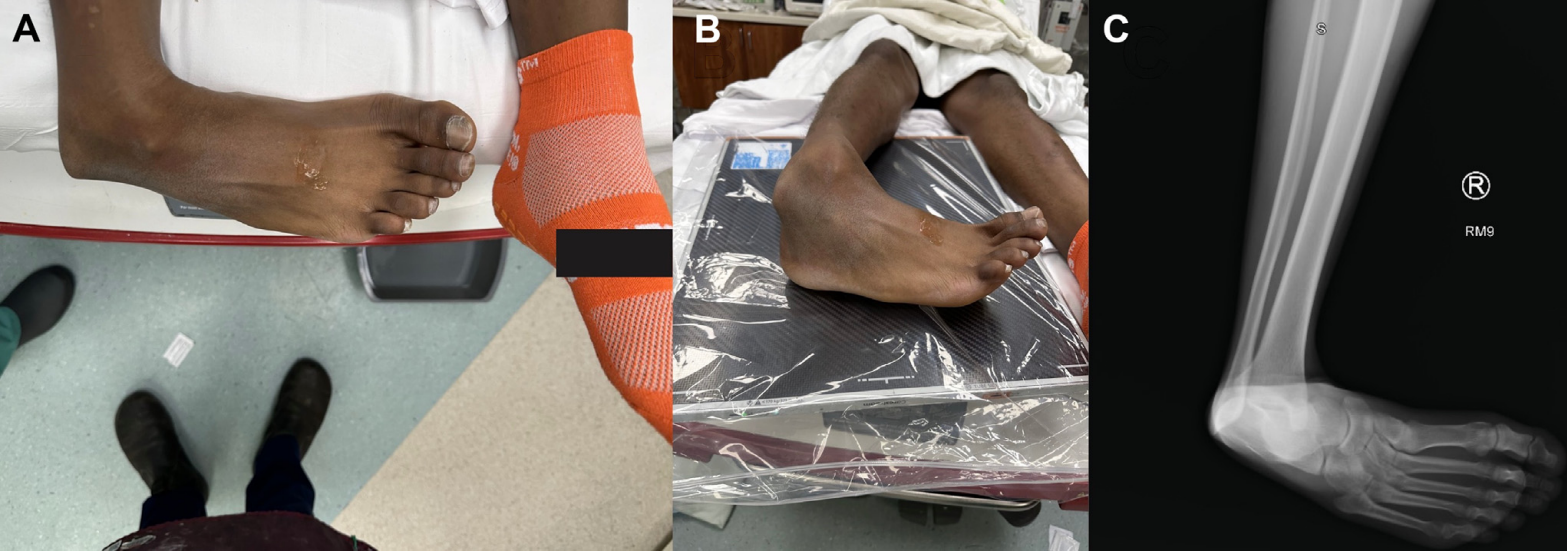
Visible deformity of the ankle with the foot positioned posterior to the ankle mortise with tenting of the skin (A, B). Anteroposterior radiograph of the right ankle showing a posterior ankle dislocation without fracture (C).

**Figure 2:**
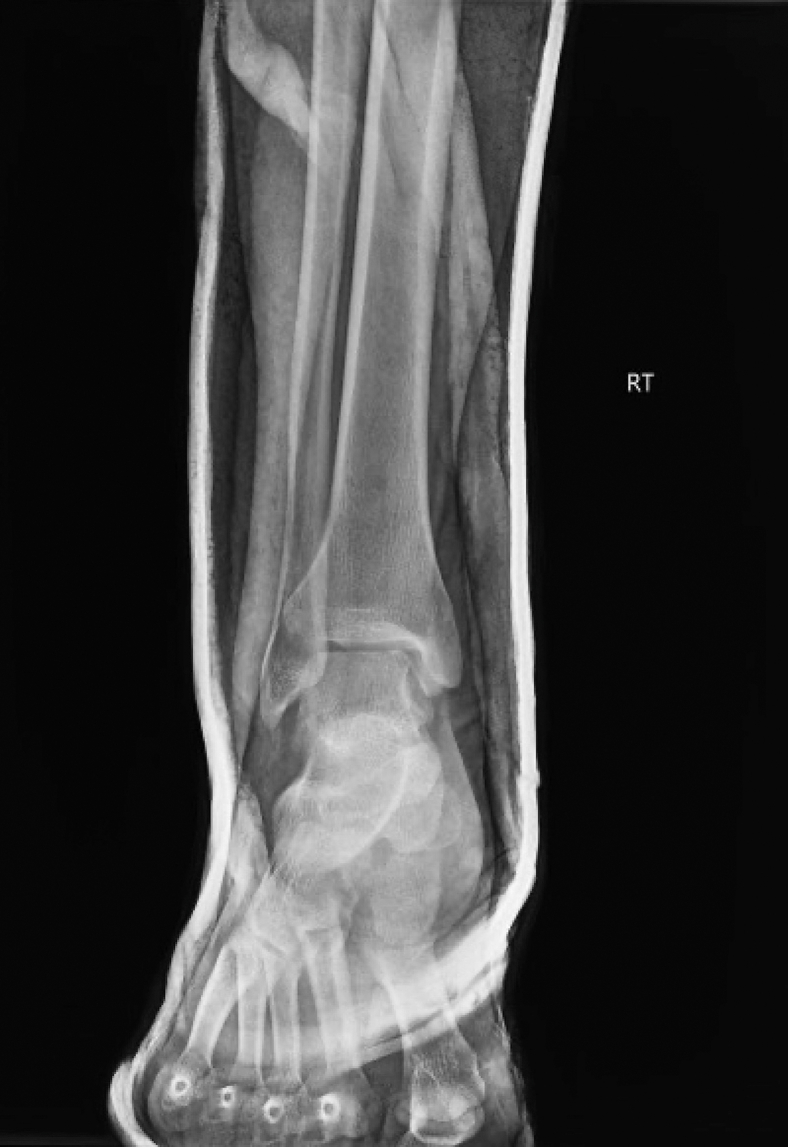
Postreduction, anteroposterior radiograph of the ankle showing closed reduction of a right-sided, posterior ankle dislocation.

A 21-year-old presented with right ankle pain following an injury sustained at an indoor trampoline park. The splint placed by the transporting paramedic was removed, which revealed a deformity at the ankle without wounds or pallor (Fig 1A, B). Distal pulses were confirmed with hand-held Doppler. Anteroposterior radiograph of the right ankle showed a posterior ankle dislocation without fracture (Fig 1C). The ankle was reduced under sedation, as confirmed by a postreduction Anteroposterior radiograph of the ankle (Fig 2) and stabilized with a posterior short leg splint with an ankle stirrup. Orthopedics was consulted and recommended discharge after computed tomography of the ankle without contrast. The computed tomography questioned a tiny osseous density adjacent to the medial malleolus with an unknown donor site but no overt fracture. During the 2-week follow-up, the splint was removed, and the patient was transitioned to a walking boot with a plan for physical therapy.

## 2 Diagnosis: Ankle Dislocation Without Fracture

Ankle dislocations occur with disruption of the tibiotalar joint and are described by the relationship of the talus to the tibia.^1^^,^^2^ The majority are posterior and often have an associated malleolar fracture.^1^^,^^2^ An ankle dislocation without concomitant fracture is rare due to the strength of the surrounding ligaments, and the estimated incidence of ankle dislocations without fracture is 0.065% of all presentations.^3^ The mechanism of a posterior dislocation is when the tibiotalar joint is maximally plantar-flexed with an axial load and forced inversion of the foot.^3^ This often disrupts the anterior talofibular and calcaneofibular ligaments.^4^

## Funding and Support

By *JACEP Open* policy, all authors are required to disclose any and all commercial, financial, and other relationships in any way related to the subject of this article as per ICMJE conflict of interest guidelines (see www.icmje.org). The authors have stated that no such relationships exist.
